# Recent advances in the diagnosis and management of pre-eclampsia

**DOI:** 10.12703/b/9-10

**Published:** 2020-11-16

**Authors:** Alice Hurrell, Kate Duhig, Brooke Vandermolen, Andrew H Shennan

**Affiliations:** 1 Women’s Health Academic Centre, King’s College London, Westminster Bridge Road, London, SE1 7EH, UK

**Keywords:** pre-eclampsia, diagnosis, placental growth factor, angiogenic biomarkers

## Abstract

Pre-eclampsia is an elusive condition to diagnose and a complex disease to manage. There have been recent developments in prediction, prevention, diagnosis, and management. Risk modelling has been used to identify women at highest risk of developing pre-eclampsia as well as predicting maternal adverse outcomes in confirmed disease. New evidence has shown that aspirin prophylaxis significantly reduces early onset pre-eclampsia as well as preterm birth. The criteria for the diagnosis of pre-eclampsia are evolving, and proteinuria is no longer a pre-requisite to make a diagnosis. Angiogenic biomarker testing accelerates diagnosis as well as minimises adverse maternal outcomes and has been incorporated into national guidelines. Emerging evidence demonstrates that expedited delivery in late preterm pre-eclampsia may be protective against maternal adverse outcomes but increase the risk of neonatal unit admission. Both women and their offspring are at increased risk of long-term health complications following pre-eclampsia, and it is important that postnatal health is optimised. This article summarises recent developments in the field of pre-eclampsia research, evaluating the impact on clinical care for women at risk of, or with suspected or confirmed, pre-eclampsia.

## Introduction

Hypertensive disorders of pregnancy complicate 10% of all pregnancies and are estimated to cause 40,000 maternal deaths worldwide each year^1,2^. Pregnancies complicated by pre-eclampsia show an increase in maternal and perinatal morbidity and mortality. The International Society for the Study of Hypertension in Pregnancy published updated guidance on the diagnosis and management of hypertensive disorders of pregnancy in 2018. The revised definition of pre-eclampsia is *de novo* hypertension after 20 weeks’ gestation with one or more of proteinuria, maternal organ dysfunction (including renal, hepatic, haematological, or neurological features), or foetal growth restriction^3^. The classification of hypertensive disorders is shown in Table 1. It is an important change that this definition does not require the presence of proteinuria to make a diagnosis of pre-eclampsia. These broader diagnostic criteria will appropriately increase the number of women assessed with suspected pre-eclampsia, resulting in a subsequent increase in the obstetric workload. It is challenging to estimate the number of women presenting with suspected pre-eclampsia, but this has been estimated at 10% of the pregnant population^4^.

**Table 1.** Classification of hypertensive disorders of pregnancy^10^.

	American College of Obstetricians and Gynecologists^11,12^	International Society for the Study of Hypertension in Pregnancy^3^	National Institute for Health and Care Excellence^6^
**Chronic** **hypertension**	BP ≥140/≥90 mmHg, pre-dating the pregnancy or before 20 weeks’ gestation	BP ≥140/≥90 mmHg, pre- dating the pregnancy or before 20 weeks’ gestation	BP ≥140/≥90 mmHg present at the booking visit or before 20 weeks’ gestation, or if the woman is already taking anti-hypertensive medication when referred to maternity services
**Gestational** **hypertension**	New-onset hypertension ≥140/≥90 mmHg, after 20 weeks’ gestation, in the absence of features of pre-eclampsia	New-onset hypertension ≥140/≥90 mmHg, after 20 weeks’ gestation, in the absence of features of pre- eclampsia	New-onset hypertension ≥140/≥90 mmHg, after 20 weeks’ gestation, without significant proteinuria
**Pre-eclampsia**	New-onset hypertension ≥140/≥90 mmHg, after 20 weeks’ gestation, with at least one of the following: • Proteinuria • Renal insufficiency • Thrombocytopenia • Impaired liver function • Pulmonary oedema • New-onset headache or visual symptoms	New-onset hypertension ≥140/≥90 mmHg, after 20 weeks’ gestation, with at least one of the following: • Proteinuria • Acute kidney injury • Haematological complications • Liver involvement • Neurological complications • Uteroplacental complications (foetal growth restriction, stillbirth)	New-onset hypertension ≥140/≥90 mmHg, after 20 weeks’ gestation, with at least one of the following: • Proteinuria • Renal insufficiency • Haematological complications • Liver involvement • Neurological complications • Uteroplacental dysfunction (foetal growth restriction, abnormal umbilical artery doppler waveform analysis, stillbirth)

BP, blood pressure

## New developments in prediction and prevention

Pre-eclampsia is notoriously difficult to predict. Accurate prediction models identifying women at high risk of disease would enable targeted prophylaxis with aspirin as well as enhanced surveillance for high-risk women to mitigate adverse outcomes. Inadequate recognition of risk contributes to substandard care associated with maternal deaths^5^. Therefore, risk prediction has been a substantial focus within the field of pre-eclampsia research.

### Risk factors

The updated National Institute for Health and Care Excellence (NICE) guidance on hypertension in pregnancy recommends a list of risk factors to identify high-risk women who should be advised to take aspirin 75–150 mg daily from 12 weeks’ gestation until birth^6^. Women should take aspirin if they have one strong risk factor or more than one moderate risk factor for pre-eclampsia. These risk factors were highlighted by a large meta-analysis of clinical risk factors for pre-eclampsia, which analysed over 25 million pregnancies^7^. Additional risk factors have also been identified and are listed in Table 2^8^. Inherited susceptibility may also play a part, and a large genome-wide association study in the offspring of 4,380 cases of pre-eclampsia identified a susceptibility locus near the *FLT1* gene encoding Fms-like tyrosine kinase 1^9^.

**Table 2.** Risk factors for pre-eclampsia^6–8^.

Strong risk factors for pre-eclampsia	Moderate risk factors for pre- eclampsia	Additional risk factors for pre- eclampsia
Hypertensive disease during a previous pregnancy	First pregnancy	Raised mean arterial pressure before 15 weeks’ gestation
Chronic kidney disease	Age 40 years or older	Polycystic ovarian syndrome
Autoimmune disease, such as systemic lupus erythematosus or antiphospholipid syndrome	Body mass index of 35 kg/m^2^ or more at first visit	Urinary tract infections
Type 1 or type 2 diabetes	Family history of pre-eclampsia	*Helicobacter pylori*
Chronic hypertension	Multi-foetal pregnancy	Vaginal bleeding for at least 5 days during pregnancy
Oocyte donation		

### Risk modelling

Pre-eclampsia is challenging to predict. There have been many studies investigating multiple-marker algorithms to predict pre-eclampsia in a similar way to first-trimester aneuploidy screening. It has been demonstrated that there are significant differences in first-trimester levels of pregnancy-associated plasma protein A (PAPP-A), a disintegrin and metalloproteinase 12 (ADAM12), and placental growth factor (PlGF)^13^; placental protein 13^14^; angiopoietin 1 and 2^15^; inhibin A and Activin A; soluble endoglin and soluble fms-like tyrosine kinase-1 (sFlt-1)^16^; and human chorionic gonadotropin (hCG)^17^.

A large systematic review compared “simple” risk models for pre-eclampsia that use routinely collected maternal characteristics against “specialised” models that include specialised tests^18^. A model using parity, history of pre-eclampsia, ethnicity, chronic hypertension, and conception method achieved an area under the curve (AUC) of 0.76 (95% confidence interval [CI] 0.74–0.77) to predict early onset pre-eclampsia, which represents a modest test. Nine studies comparing simple versus specialised models in the same population reported AUCs favouring specialised models. A simple model achieved fewer false positives than a guideline-recommended risk factor list such as the NICE hypertension in pregnancy guideline, but the clinical value of different models to guide aspirin prophylaxis still needs to be determined.

Wright and colleagues recently studied the effect of two-stage screening, with a first stage screening of the whole population based on risk factors and a second stage “triple test” (comprising maternal factors, mean arterial pressure, uterine artery pulsatility index, and PlGF) for those identified as high risk^19^. The authors have previously demonstrated that their triple test is superior to risk factor-based screening and can predict 90% of early pre-eclampsia (necessitating delivery before 32 weeks’ gestation) and 75% of preterm pre-eclampsia at a screen-positive rate of 10%^20^. Using this two-stage strategy of screening would mean that only 70% of the population would require the triple test whilst achieving similar detection and screen-positive rates. This would clearly have financial benefits over intensive screening of the whole population and may be a promising area of development.

### Prophylaxis

Accurate prediction of pre-eclampsia will facilitate targeted prophylaxis with aspirin. Initial studies demonstrated that early administration of prophylactic aspirin in high-risk women prior to 16 weeks’ gestation reduced the risk of pre-eclampsia by 17%, with an 8% relative risk reduction of preterm birth and a 14% reduction in foetal and neonatal death^21^. Aspirin prophylaxis has recently been investigated in the ASPRE trial, which was a multicentre double-blind randomised controlled trial including 1,620 women (aspirin versus placebo in pregnancies at high risk for preterm pre-eclampsia)^22^. This trial demonstrated that 150 mg aspirin resulted in a 60% reduction in preterm pre-eclampsia (1.6% compared to 4.3%, adjusted odds ratio [OR] 0.38, 95% CI 0.2–0.74) and a 90% reduction in early onset pre-eclampsia, with no significant effect on term pre-eclampsia. The authors hypothesise that the larger effect size seen in their trial may be due to the higher dose of aspirin taken at night and commenced prior to 16 weeks’ gestation. Similar results were found in a recent meta-analysis^23^. Recent evidence has also emerged from the ASPIRIN study, a randomised, double-blind, placebo-controlled trial of low-dose aspirin for the prevention of preterm birth in 11,976 women from low-income countries^24^. This demonstrated a small but significant reduction in preterm birth (relative risk [RR] 0.89, 95% CI 0.81–0.98) and a larger reduction in preterm birth before 34 weeks’ gestation in women with hypertensive disorders (RR 0.38, 95% CI 0.17–0.85). Therefore, aspirin is a safe and simple strategy that is likely to have far-reaching benefit. However, non-adherence may be an underestimated problem. A study of 220 women found that 44% of women were non-adherent and that these women had significantly higher rates of early onset pre-eclampsia (OR 1.9, 95% CI 1.1–8.7), late-onset pre-eclampsia (OR 4.2, 95% CI 1.2–8.3), and intrauterine growth restriction (OR 5.8, 95% CI 1.2–10.5)^25^. Furthermore, self-reported adherence does not represent actual adherence.

Other interventions have been investigated to assess impact on pre-eclampsia, including nutritional supplements and dietary and lifestyle modifications. Some studies have suggested benefit with vitamin D supplementation^26^, but robust evidence from randomised controlled trials is lacking. A Cochrane systematic review found that high-dose calcium supplementation during pregnancy reduces the risk of pre-eclampsia and preterm birth, especially in women with a diet deficient in calcium (<600 mg/day)^27^. Calcium supplementation is not recommended in women with normal dietary calcium intake, but the World Health Organization recommends daily calcium supplementation (1.5–2 g) for pregnant women in populations with low dietary calcium intake. Supplementation with vitamins C and E has no benefit in preventing pre-eclampsia and is not recommended^28^.

## New developments in diagnosis

### Assessing hypertension and proteinuria

Pre-eclampsia is elusive to diagnose. Pre-eclampsia is *de novo* hypertension after 20 weeks’ gestation with one or more of proteinuria, maternal organ dysfunction (including renal, hepatic, haematological, or neurological features), or foetal growth restriction. Hypertension is classified as a systolic blood pressure of 140 mmHg or higher and/or a diastolic blood pressure of 90 mmHg or higher at or after 20 weeks’ gestation^3^. Approximately 25% of those with a background of chronic hypertension will develop super-imposed pre-eclampsia, which is defined as a worsening of hypertension in association with new-onset maternal organ dysfunction^3^.

Recent research has focused on whether ambulatory or home blood pressure monitoring impacts maternal outcomes in pre-eclampsia. A systematic review from 2002 concluded that there was no randomised controlled trial evidence to support ambulatory blood pressure monitoring and recommended adequately powered randomised trials to evaluate this^29^. OPTIMUM (optimising titration and monitoring of maternal blood pressure) is a randomised controlled trial of blood pressure self-monitoring during pregnancy for women with chronic hypertension, which aims to assess the feasibility and most appropriate outcome measures for a larger trial^30^. The BUMP trial (blood pressure monitoring in high-risk pregnancy to improve the detection and monitoring of hypertension) is a randomised controlled trial to determine whether self-monitoring leads to earlier diagnosis of raised blood pressure and lower mean systolic blood pressure between baseline and delivery^31^. Recruitment has finished for both trials and the results are awaited.

Additionally, assessment of proteinuria is variable. The gold standard for assessment of proteinuria was previously a 24-hour urine collection. However, this was logistically challenging and prone to error^32^. The DAPPA study (diagnostic accuracy in pre-eclampsia using proteinuria assessment) recruited 959 women, of whom 417 had severe pre-eclampsia, and compared spot protein:creatinine ratio (PCR) and spot albumin:creatinine ratio (ACR) against the reference standard of 24 hour urine collection^33^. They found that the diagnostic accuracy of PCR and ACR was similar to a 24-hour urine collection and that ACR had a significantly higher sensitivity of 99% compared to 90% with PCR. Therefore, NICE recommends dipstick screening for proteinuria and, if positive (1+ or more), then ACR or PCR should be used to quantify proteinuria^6^.

### Novel methods of diagnosis

The diagnosis of pre-eclampsia is complex, particularly on a background of medical co-morbidities. Hypertension, proteinuria, and biochemical and haematological abnormalities are tertiary, downstream features of established disease, which may be absent even in women presenting with eclampsia^34^. There is a need for better methods of diagnosis and risk stratification of women at risk of pre-eclampsia. Angiogenic biomarkers are closely linked to the pathophysiology of pre-eclampsia, and abnormalities in angiogenic biomarker concentrations such as PlGF and sFlt-1 have been identified up to 10 weeks before the clinical onset of disease^35^. The role of angiogenic biomarkers in diagnosis and risk stratification in suspected pre-eclampsia has been investigated, and there have been recent developments.

**Figure 1.**
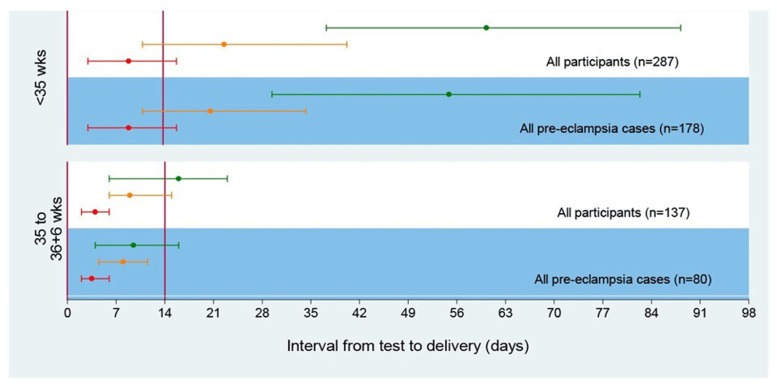
Time to delivery (median, interquartile range) stratified by PlGF concentration for all participants and for pre-eclampsia cases^29^. Red line indicates very low PlGF (<12 pg/ml), orange line indicates low PlGF (< fifth centile), and green line indicates normal PlGF (≥ fifth centile). PlGF, placental growth factor. This figure was reproduced from Duhig *et al*.^41^ under the terms of the Creative Commons Attribution 4.0 International license (CC-BY 4.0).

Chappell and colleagues investigated the diagnostic accuracy of PlGF in the prospective, multicentre PELICAN study, which included 625 women^36^. Low PlGF concentrations demonstrated high sensitivity (0.96, 95% CI 0.89–0.99) and negative predictive value (0.98, 95% CI 0.93–0.995) for diagnosing pre-eclampsia necessitating delivery within 14 days in women with suspected pre-eclampsia before 35 weeks’ gestation (Figure 1)^36^. PlGF outperformed all other tests commonly used to diagnose pre-eclampsia (systolic blood pressure, diastolic blood pressure, alanine transaminase, urate, and dipstick proteinuria). Prediction of short-term outcome in pregnant women with suspected pre-eclampsia (PROGNOSIS) was a prospective, multicentre observational study investigating the clinical utility of a sFlt-1:PlGF ratio, with 500 women included in a development cohort to determine a ratio cut-off and 550 women as a validation cohort^37^. sFlt-1:PlGF ratios of 38 or lower have a high negative predictive value (99.3%, 95% CI 97.9–99.9) and an 80% sensitivity (95% CI 51.9–95.7) for diagnosing pre-eclampsia within 1 week. The positive predictive value of an sFlt-1:PlGF ratio above 38 for a diagnosis of pre-eclampsia within 4 weeks was 36.7% (95% CI 28.4–45.7), and sensitivity was 66.2% (95% CI 54.0–77.0). The authors conclude that a high negative predictive value is crucial in the evaluation of suspected pre-eclampsia, as failure to detect imminent disease could have important consequences for the woman or foetus.

More recently, the PlGF test and sFlt-1:PlGF ratio have been assessed in further studies. The PETRA study (pre-eclampsia triage by rapid assay) was a large prospective cohort study in North America^38^. This found that a low PlGF concentration of <100 pg/ml was significantly associated with preterm delivery as well as adverse neonatal outcomes (9.2% compared to 0.8%, adjusted RR 17.2, 95% CI 5.2–56.3) and maternal outcomes (6.2% compared to 1.9%, adjusted RR 3.6, 95% CI 1.7–8.0)^39^. The authors conclude that PlGF may be useful for risk stratification for women presenting with suspected pre-eclampsia. The PARROT trial (PlGF to assess and diagnose hypertensive pregnant women: a stepped wedge trial) was a stepped-wedge cluster-randomised controlled trial of revealed versus concealed PlGF testing implemented alongside a clinical management algorithm^40^. This trial enrolled 1,023 women and demonstrated that time to diagnosis was reduced from 4.1 to 1.9 days, and severe adverse maternal outcomes were reduced from 5.4 to 3.8% (adjusted OR 0.32, 95% CI 0.11–0.96). There was no difference in gestational age at delivery or adverse perinatal outcomes. Finally, the INSPIRE trial (a prospective, randomised interventional study evaluating the short-term prediction of pre-eclampsia/eclampsia in pregnant women with suspected pre-eclampsia) evaluated the use of the sFlt-1:PlGF ratio^42^. This trial found no difference in the primary endpoint of hospitalisation within 24 hours of testing but demonstrated that a higher proportion of women were admitted who subsequently developed pre-eclampsia over the following 7 days, demonstrating more appropriate use of resources.

Initial economic models predicted that PlGF-based testing may afford a cost-saving of between £330 and £1,032 per woman tested^43,44^. In a secondary cost-saving analysis of the PARROT trial, PlGF testing resulted in a total cost-saving of £149 per woman (based on £70 per PlGF test)^45^. This more conservative estimate is likely because of an appropriate redistribution of resources rather than an overall reduction in resources as anticipated by hypothetical analyses. In view of the evidence for clinical and cost benefit, the updated NICE Guideline on Hypertension in Pregnancy recommends a single PlGF-based test at the time of presentation with suspected preterm pre-eclampsia between 20 and 34^+6^ weeks’ gestation^6,46^.

Unfavourable angiogenic biomarker profiles are particularly linked to adverse perinatal outcomes, including foetal death and severe intrauterine growth restriction. In a study of 412 women with suspected pre-eclampsia, women with pre-eclampsia and adverse outcomes had lower PlGF and higher sFlt-1:PlGF ratio than women without adverse outcomes (*P* <0.0001)^47^. A case-control study of 11 cases of foetal death and 829 controls found that an angiogenic index-1 value <2.5^th^ centile was associated with a 29-fold increase in the risk of foetal death^48^. Another study of 314 pregnant women with suspected small-for-gestational-age foetuses (estimated foetal weight <10^th^ centile) found that angiogenic biomarkers could identify the majority of women who subsequently developed pre-eclampsia or indicated preterm delivery, with AUC greater than 80%^49^. However, in other studies, there have been important false negatives, including stillbirth^50^.

### Novel methods of risk prediction

There are now externally validated risk prediction models available to predict adverse maternal outcomes once pre-eclampsia has been diagnosed and to guide clinical management, including timing of delivery, antenatal steroids, magnesium sulphate, and transfer to high-level care. The fullPIERS model is intended for use at any time in pregnancy and predicts adverse outcomes in the next 48 hours^51^. The fullPIERS calculator is available online and is based on gestational age, chest pain or dyspnoea, oxygen saturation, creatinine, platelets, and aspartate aminotransaminase (AST) or alanine aminotransaminase (ALT). The PREP-S prediction model is intended for use up to 34 weeks’ gestation and provides robust estimates of the overall risk of adverse maternal outcomes^52^. The PREP-S model requires maternal age, gestational age, medical co-morbidities, PCR, urea, creatinine, ALT, platelets, systolic blood pressure, pulse oximetry, presence of exaggerated tendon reflexes, and treatment with anti-hypertensive drugs or magnesium sulphate. However, neither of these models predict adverse perinatal outcomes.

## Management

### Blood pressure

The revised NICE Guideline recommends offering treatment for hypertension in pregnancy if systolic blood pressure is sustained above 140 mmHg or diastolic blood pressure is sustained above 90 mmHg. Once anti-hypertensive treatment has been started, the target blood pressure is 135/85 mmHg^6^. This is an important change from previous practice, when treatment was recommended if blood pressure exceeded 150/100 mmHg and reflects evidence from CHIPS (Control of Hypertension in Pregnancy Study)^53^. This trial was an international multicentre randomised controlled trial comparing “tight” blood pressure control (target diastolic blood pressure 85 mmHg) to “less tight” blood pressure control (target diastolic blood pressure 100 mmHg) in women with non-severe non-proteinuric maternal hypertension. A total of 981 women were randomised, and the results demonstrated that those with “tight” control were less likely to experience severe maternal hypertension (*P* <0.001), without any effect on adverse perinatal outcome or birthweight <10^th^ centile. Severe maternal hypertension was significantly associated with the primary composite outcome of perinatal loss or high-level neonatal care for >48 hours as well as serious maternal complications. As this study included women with non-proteinuric hypertension, the results should be extrapolated to pre-eclampsia with caution, but there may be benefit in tighter control of blood pressure.

The revised NICE guidance recommends labetalol as the first-line treatment for hypertension in pregnancy, with nifedipine recommended if labetalol is not suitable and methyldopa recommended if neither labetalol or nifedipine are suitable or tolerated^6^. It is vital that women are provided with information on the benefits of treatment and the side effects of the various options for treatment to enable shared decision making and informed choice.

### Delivery

Both NICE in the United Kingdom and the American College of Obstetricians and Gynecologists recommend delivery at 37 weeks’ gestation for women with confirmed pre-eclampsia. Before 34 weeks’ gestation, expectant management is advised, as iatrogenic preterm delivery before 34 weeks’ gestation is associated with worse neonatal adverse outcomes (respiratory distress syndrome RR 2.3, 95% CI 1.39–3.81, and necrotising enterocolitis RR 5.54, 95% CI 1.04–29.56)^54^. Recent research has been investigating the optimum time to deliver between 34 and 37 weeks’ gestation to prevent morbidity for women and their babies.

The HYPITAT-II randomised controlled trial (immediate delivery versus expectant monitoring for hypertensive disorders of pregnancy between 34 and 37 weeks of gestation) investigated the effect of immediate delivery compared to expectant monitoring on maternal and neonatal outcomes in women with hypertensive disorders in late preterm pregnancy^55^. A total of 703 women were enrolled from 51 hospitals in the Netherlands. The results demonstrated that immediate delivery (by induction or elective caesarean section) was associated with a non-significant reduction in a composite of severe maternal adverse outcomes (RR 0.36, 95% CI 0.12–1.11; *P* = 0.067). However, there was an increased risk of neonatal respiratory distress syndrome in the immediate delivery group (RR 3.3, 95% CI 1.4–8.2; *P* = 0.005), and thus the authors conclude that routine expedited delivery does not seem to be justified. Two-year infant follow up from the HYPITAT-II trial showed a significant increase in neurodevelopmental delay, but this was not evident at 5-year follow up^56,57^.

The PHOENIX randomised controlled trial (pre-eclampsia in hospital: early induction or expectant management) investigated planned delivery versus expectant management in women diagnosed with late preterm pre-eclampsia between 34 and 36^+6^ weeks’ gestation^58^. This trial of 901 women demonstrated that planned delivery was associated with a significant reduction in adverse maternal outcomes (65% compared to 75%, adjusted RR 0.86, 95% CI 0.79–0.94, *P* = 0.0005) and a significant increase in neonatal unit admissions for prematurity but without any other indicators of neonatal morbidity. The authors conclude that this trade-off should be discussed with women with late-preterm pre-eclampsia to enable informed shared decision making. This larger trial suggests maternal benefit in earlier delivery from 34 weeks in all women with pre-eclampsia when combined with the previous data demonstrating beneficial trends. A meta-analysis is under way. Evidence from a low-income setting is required where both potential harms and benefits are considerably greater and where the vast burden of pre-eclampsia disease is found.

### Long-term complications

Pre-eclampsia has long-term health implications for women^59^. Numerous high-quality studies have now firmly established that pre-eclampsia increases lifetime risk of cardiovascular disease. A meta-analysis of over 3 million women demonstrated increased risk of vascular disease, with a RR of 3.7 for hypertension and 2.16 for ischaemic heart disease^60^. This effect is multiplied after recurrent pre-eclampsia, with a hazard ratio of 2.04 for all-cause mortality, 5.10 for stroke, and 3.30 for ischaemic heart disease in a retrospective cohort study of 57,384 women^61^. Follow up from the HYPITAT trial showed that almost half of women with early onset pre-eclampsia developed hypertension over 2–5 years post-delivery compared to 39% and 25% of women in the pregnancy-induced hypertension and late-onset pre-eclampsia groups, respectively^62^. Long-term follow up from PHOENIX has also been undertaken and is pending.****A study of 31 women with a history of early onset pre-eclampsia found subclinical impairment of left ventricular function 12 years after pre-eclampsia compared to women with late-onset pre-eclampsia or normotensive pregnancies (n = 62)^63^. As well as links with long-term cardiovascular health, pre-eclampsia has also been associated with peripartum cardiomyopathy, and the latter has similarly been associated with an imbalance of angiogenic factors^64^.

Long-term risks impact not only women but also their offspring. A meta-analysis of 53,029 individuals demonstrated that *in utero* exposure to pre-eclampsia resulted in 5.17 mmHg greater systolic blood pressure and 4.06 mmHg greater diastolic blood pressure in childhood^65^. Similar results were demonstrated in a 20-year prospective follow-up cohort of 2,868 young adults, which found that exposure to hypertension *in utero* increased the risk of hypertension in offspring by 2.5 times^66^.

## Conclusions

Important evidence is still emerging to improve the diagnosis and management of this complex disease. Screening models using specialised tests show promise, and aspirin may have far-reaching effects in reducing both early onset pre-eclampsia and preterm birth. New evidence is awaited regarding the impact of home blood pressure monitoring on severe hypertension and adverse outcomes. The CHIPS trial demonstrated better outcomes with tight blood pressure control in pregnancy hypertension, and this has been incorporated into national guidance^6^. Angiogenic biomarkers accelerate diagnosis and minimise adverse maternal outcomes when used in the assessment of suspected disease. Their use will enable risk stratification and appropriate resource redistribution and are similarly recommended in updated national guidelines. There is evidence regarding timing of delivery between 34 and 37 weeks’ gestation in confirmed pre-eclampsia, and shared decision-making with women regarding anti-hypertensive medication and timing of delivery is vital.

The most devastating complication in pre-eclampsia is maternal death. The MBRACE report (mothers and babies: reducing risk through audits and confidential enquiries across the UK) found that hypertension-related maternal deaths are at the lowest rate ever^67^. It is a triumph of modern obstetrics that there is fewer than one maternal death from hypertensive disorders of pregnancy per million births. Optimising management to improve perinatal outcomes remains a challenge, particularly in a global setting.

## Abbreviations

ACR, albumin:creatinine ratio; ALT, alanine aminotransaminase; AUC, area under the curve; CHIPS, Control of Hypertension in Pregnancy Study; CI, confidence interval; NICE, National Institute for Health and Care Excellence; OR, odds ratio; PCR, protein:creatinine ratio; PlGF, placental growth factor; RR, relative risk; sFlt-1, soluble fms-like tyrosine kinase 1.
